# Supplementary material for: Rapid and efficient optical tissue clearing for volumetric imaging of the intact and injured spinal cord in mice
Source: Front Neurosci. 2025 Jun 13;19:1601360. doi: 10.3389/fnins.2025.1601360 (PMC12202625; doi:10.3389/fnins.2025.1601360)
Supplement: Supplementary file 1 [file Supplementary_file_1.zip › Agarose Mounting Mold and Peg/Agarouse Mold and Peg 3D Printing Instructions.docx]

**Material:** 

- Z7 Lightsheet Mounting Peg: **Polypropylene***

- Will need to lay packing tape on the bed for the material to stick

- Agarose Mold: PLA

**Layer Height:** 

- Peg: 0.12mm

- Mold: 0.12mm

**Supports:** None
