# Supplementary material for: Rapid and efficient optical tissue clearing for volumetric imaging of the intact and injured spinal cord in mice
Source: Front Neurosci. 2025 Jun 13;19:1601360. doi: 10.3389/fnins.2025.1601360 (PMC12202625; doi:10.3389/fnins.2025.1601360)
Supplement: Supplementary file 1 [file Supplementary_file_1.zip › Spinal Cord Straightener/Spinal Cord Straightener 3D Printing Instructions.docx]

**3D Printing Instructions for the Spinal Cord Straightener**

**Material:** Polypropylene

**Layer Thickness:** 0.12mm

**Supports:** None

**Print Bed:** Stick a layer of packing tape on the print bed. This will allow the polypropylene to adhere to the bed.

Print 2 of the “Cord Straightener Body” and 1 of the “Strap_withHole”
